# Functional and Morphological Changes in Endocrine Pancreas following Cola Drink Consumption in Rats

**DOI:** 10.1371/journal.pone.0118700

**Published:** 2015-03-19

**Authors:** Matilde Otero-Losada, Gabriel Cao, Julián González, Angélica Muller, Graciela Ottaviano, Christopher Lillig, Francisco Capani, Giuseppe Ambrosio, José Milei

**Affiliations:** 1 Instituto de Investigaciones Cardiológicas, Universidad de Buenos Aires, Consejo Nacional de Investigaciones Científicas y Técnicas, ININCA.UBA.CONICET, Buenos Aires, Argentina; 2 Institut für Biochemie und Molekularbiologie, Universitätsmedizin Greifswald KdöR, Ernst Moritz Arndt Universität, Greifswald, Germany; 3 Università di Perugia, Cardiologia e Fisiopatologia Cardiovascolare, Perugia, Italy; Kobe University, JAPAN

## Abstract

**Aim:**

We report the effects of long-term cola beverage drinking on glucose homeostasis, endocrine pancreas function and morphology in rats.

**Methods:**

Wistar rats drank: water (group W), regular cola beverage (group C, sucrose sweetened) or “light” cola beverage (group L, artificially sweetened). After 6 months, 50% of the animals in each group were euthanized and the remaining animals consumed water for the next 6 months when euthanasia was performed. Biochemical assays, insulinemia determination, estimation of insulin resistance (HOMA-IR), morphometry and immunohistochemistry evaluations were performed in pancreas.

**Results:**

Hyperglycemia (16%, p<0.05), CoQ_10_ (coenzyme-Q_10_) decrease (−52%,p<0.01), strong hypertriglyceridemia (2.8-fold, p<0.01), hyperinsulinemia (2.4 fold, p<0.005) and HOMA-IR increase (2.7 fold, p<0.01) were observed in C. Group C showed a decrease in number of α cells (−42%, p<0.01) and β cells (−58%, p<0.001) and a moderate increase in α cells’ size after wash-out (+14%, p<0.001). Group L showed reduction in β cells’ size (−9%, p<0.001) and only after wash-out (L_12_) a 19% increase in size (p<0.0001) with 35% decrease in number of α cells (p<0.01). Groups C and L showed increase in α/β-cell ratio which was irreversible only in C (α/β = +38% in C_6_,+30% in C_12_, p<0.001vs.W_6_). Regular cola induced a striking increase in the cytoplasmic expression of Trx1 (Thioredoxin-1) (2.25-fold in C_6_ vs. W_6_; 2.7-fold in C_12_ vs. W_12,_ p<0.0001) and Prx2 (Peroxiredoxin-2) (3-fold in C_6_ vs. W_6_; 2-fold in C_12_ vs. W_12,_ p<0.0001). Light cola induced increase in Trx1 (3-fold) and Prx2 (2-fold) after wash-out (p<0.0001, L_12_ vs. W_12_).

**Conclusion:**

Glucotoxicity may contribute to the loss of β cell function with depletion of insulin content. Oxidative stress, suggested by increased expression of thioredoxins and low circulating levels of CoQ_10_, may follow sustained hyperglycemia. A likely similar panorama may result from the effects of artificially sweetened cola though via other downstream routes.

## Introduction

We have reported the pathophysiological alterations observed in normal rats [1, 2] and in atherosclerosis prone mice [3, 4] following cola beverages’ long-term drinking. Both sugar-sweetened and artificially sweetened cola beverages caused alterations. Notwithstanding that only sugar-sweetened cola consumption resulted in typical metabolic syndrome alterations (obesity, hypertension, hyperglycemia and dyslipidemia) [5], artificially sweetened cola drinking resulted in alterations as well. Moreover in atherosclerotic prone mice, light cola accelerated the progression of aortic wall injury to a greater extent compared to regular cola [4]. Along with the changes observed in rats, a pro-oxidative metabolism was suggested based on the decrease in plasma levels of coenzyme Q_10_ (CoQ_10_) and the increase in the ratio of pro-oxidative to antioxidants compounds [2]. Most interestingly, the levels of CoQ_10_ were inversely correlated to triglyceridemia and the development of left ventricular hypertrophy observed in cola drinking rats [1, 2] suggesting mutual relationship.

It is known that sustained hyperglycemia may lead to insulin resistance and type 2 diabetes as well depending on genetic and epigenetic background [6, 7]. Type 2 diabetes has been associated with oxidative stress and a generalized inflammatory condition [8]. Metabolic syndrome and diabetes are known risk factors for cardiovascular disease, which is the leading cause of death in modern Western societies and soft drink consumption has been related to obesity and increased risk of metabolic syndrome. Individuals consuming >500mL soft drink per day had a higher prevalence of metabolic syndrome than those consuming <1 drink per day [5]. It is widely accepted that sugar-sweetened soft drink consumption increases the risk of metabolic disorders. Unlike sugar, artificial sweeteners (AS) are usually considered safe and beneficial owing to their low caloric content and artificially sweetened beverages are marketed as low-calorie substitutes to prevent beverages-associated weight gain [9].While it is undeniable that AS do not add extra calories, they may pose to other risks instead.

This paper addresses the effects of cola drink consumption on endocrine pancreas function and morphology regarding glucose homeostasis in rats. The contribution of cell proliferation, apoptosis and/or oxidative stress to the observed changes was evaluated in insular α and β cells in pancreas.

## Material and Methods

The experiment was conducted in accordance with the recommendations of the Weatherall report, "The use of non-human primates in research." The committee of Ethics in Animal Research of the Instituto de Investigaciones Cardiológicas (ININCA) and the Institutional Animal Care and Use Committee (IACUC) of the Faculty of Medicine of the University of Buenos Aires (CICUAL, Institutional Committee for the Care and Use of Laboratory Animals) approved the study. Animals were housed at the ININCA facilities (21±2°C, at 12-h light-dark cycles 7am-7pm) and were fed a commercial chow (16%-18% protein, 0.2 g % sodium (Cooperación, Buenos Aires, Argentina) *ad libitum*. Animal handling, maintenance and euthanasia procedures were performed according with international recommendations [10].

### Experimental protocol

Forty-eight male Wistar rats were randomly distributed in 3 groups, which were respectively assigned to different treatments according to beverage (as the only liquid source, *ad libitum*): W (water), regular cola (C) (commercially available sucrose-sweetened carbonated drink, Coca-Cola, Argentina) and LightCola (L) (commercially available low calorie aspartame-sweetened carbonated drink, Coca-Cola Light, Argentina). Six months after the beginning of the study (end of treatment) 50% of the animals in each group (C, L and W) were euthanized by subtotal exsanguination under anesthesia (sodium thiopental 40 mg/kg, i.p.). The remaining animals went on drinking tap water (group W) or were switched to tap water (groups C and L) *ad libitum* for the following 6 months (wash-out period: months 7 to 12 after beginning of the study). Rats were weighed weekly. Food and drink consumption were assessed twice a week. Biochemical assays were performed at baseline, 6 months (treatment) and 12 months (wash-out). Histopathological data were obtained at the end of treatment (6 months) and after the wash-out period (12 months).

According to company specifications Coca Cola^TM^ is a carbonated water solution containing (approximate %): 10.6 g carbohydrates, sodium 7 mg, caffeine 11.5 mg, caramel, phosphoric acid, citric acid, vanilla extract, natural flavorings (orange, lemon, nutmeg, cinnamon, coriander, etc), lime juice and fluid extract of coca (Erythroxylon novogranatense). As far as nutritional information is concerned the only difference between regular and light cola is the replacement of carbohydrates with non-nutritive sweeteners (aspartame + acesulfame K) in the latter.

Soft drinks had carbon dioxide content largely removed by vigorous stirring using a stirring plate and placing a magnetic bar in a container filled with the liquid prior to being offered to the animals at room temperature.

### Biochemical determinations

Plasma aliquots of blood collected from the tail vein after 4-hour fasting were used to measure the concentration of glucose and triglycerides by enzymatic colorimetric assays using commercially available kits (Sigma-Aldrich, USA) according to manufacturer’s instruction [11]. Plasma concentration of the lipophilic antioxidant ubiquinone-10 (2,3 dimethoxy-5 methyl-6-decaprenyl benzoquinone-10, coenzyme Q_10_) was measured using RP-HPLC with UV detection at wavelength 275nm [12]. Insulinemia was measured by ELISA (Mercodia Rat Insulin ELISA, Catalog nr 10-1250-01).

### HOMA-IR (homeostatic model assessment of insulin resistance) index

The HOMA-IR index was used to estimate insulin resistance and was calculated using the validated formula for Wistar rats [13, 14]: HOMA-IR = (I x G) /k, where I = fasting insulinemia, G = fasting glycemia and k is a constant value (k = 405 if G is expressed in mg/dL and k = 22.5 if G is expressed in mmol/L).

### Quantitative Morphology

At the above indicated times euthanasia was practiced in 50% of the animals in each experimental group. The whole pancreas was weighed and fixed in buffered 4% formaldehyde solution for 24h at room temperature, dehydrated in alcohols, cleared in xylene and embedded in paraffin.

For light microscopy, a Nikon Eclipse 50i microscope (Nikon Corporation, Tokyo, Japan), equipped with a digital camera (Nikon Coolpix S4) and the Image-Pro Plus image processing software version 6.0 (Media Cybernetics, Silver Spring, Maryland, USA) were used. Thus, 12 to 16 fields of view were obtained by systematic uniform random sampling of pancreas.

For stereological analysis 3 μm width sections were cut from tissue blocks, stained with hematoxylin-eosin and used for immunohistochemistry. An orthogonal grid with 300 test points representing an area of 6.7 10^4^μm^2^ (objective lens magnification: 40 X) was used. Points were projected onto the fields of view and the number of points hitting structures of interest was counted. The point-counting method was used to estimate [15]: α/β-cell ratio, Langerhans islet area [A_islet_, 10^4^ μm^2^] and immunohistochemical staining areas for glucagon [A_α_-cells (10^4^μm^2^)/islet], insulin [A_β-cells_ (10^4^μm^2^)/islet], caspase-3 [A_caspase-3_ (10^4^μm^2^)/islet], PCNA (Proliferating cell nuclear antigen) [A_PCNA_ (10^4^μm^2^)/islet], thioredoxin-1 (Trx1) [A_Trx1_ (10^4^μm^2^)/islet] and peroxiredoxin-2 (Prx2) [A_Prx2_ (10^4^μm^2^)/islet].

### Immunohistochemistry

Alpha and beta pancreatic cells were evaluated in dewaxed sections using specific antibodies (mouse monoclonal anti-Glucagon and anti-Insulin antibodies, Sigma-Aldrich Corp., St. Louis, MO USA). A rabbit polyclonal antibody against Caspase-3 (1:100, Santa Cruz Biotechnology, Inc., Santa Cruz, CA, USA) and a mouse monoclonal antibody anti-PCNA (Biogenex Laboratories, CA, USA) were respectively used to estimate apoptosis and cellular proliferative activity. Rabbit polyclonal antibodies against thioredoxin-1 and peroxiredoxin-2(Trx1 and Prx2, 1:200, provided by Ch. Lillig) were used to evaluate oxidative stress. Before staining, sections were deparaffinized and incubated in 3% hydrogen peroxide for 10 min to quench endogenous peroxidase. After washing 3 times in PBS, nonspecific antibody binding sites were blocked with 10% normal goat serum in PBS. Sections were incubated with the primary antibodies diluted in blocking solution at 4°C overnight. Negative controls were incubated with 10% goat serum in PBS. Sections were then washed 3 times in PBS and subsequently incubated with a biotinylated secondary anti-mouse or anti-rabbit antibody diluted 1:500 (Dako, Glostrup, Denmark) for 60 min at room temperature. Immunohistochemical staining was obtained performed using a biotinylated-streptovidin-peroxidase complex (Dako Universal LSAB™+ Kit/HRP-K0690) with DAB (3,3-Diaminobenzidin)-chromogen (Dako-K3468) as detection system according to manufacturer recommendations.

### Statistical analysis

Data were analyzed by two-way ANOVAs followed by post-hoc tests (Bonferroni multiple t-test) in order to evaluate between-groups’ differences. Pearson correlation test was used to evaluate associations between variables. Statistical significance was set at p≤0.05 and SPSSversion 15.0 software was used to analyze data.

## Results

### Nutritional considerations

Regular cola drinking for 6 months (C_6_) caused an increase in liquid and caloric intake (+69%, p<0.001 and +12%, p<0.05 respectively) and a decrease in food intake (−31%, p<0.001). These changes were observed even after the wash-out period (p<0.05 vs. W_12_): +59% in drinking volume and −31%in food intake (Table 1). Body weight increased after regular cola drinking for 6 months (7%, p<0.001vs. W_6_). Age was independently associated with an increase in body weight (W_12_+10%, p<0.01vs. W_6_) (Table 1).

**Table 1:** Nutritional data and body weight.

	Treatment			Wash-out		
Group (n)	W_6_ (16)	C_6_ (16)	L_6_ (16)	W_12_ (16)	C_12_ (16)	L_12_ (16)
**Body Weight (g)**	626±8	669±9**	630±9	689±10#	703±27	699±61
**Liquid intake (ml/100 g BW)**	8.7±1.2	14.7±2.8***	8.5±1.7	7.1±1.1	11.3±1.8*** #	6.5±1.5
**Solid intake (g/100 g BW)**	4.9±0.6	3.4±0.6***	5.1±0.4	5.5±0.6	3.8±0.6*	4.9±0.6*
**Liquid energy (Kcal/100 g BW)**	0	6.17±0.5***	0.09±0.00	0	0	0
**Solid energy (Kcal/100 g BW)**	14.7±1.1	10.2±1.3	15.3±0.9	16.5±1.0	11.4±0.9	14.7±0.9
**Total energy (Kcal/100g BW)**	14.7±1.1	16.4±1.2**	15.4±0.9	16.5±1.5	11.4±1.1#	14.7±0.9

Treatment: water (W), regular (C), or light cola drinking (L) for 6 months. Wash-out: switch from both caloric and non-caloric soft drink to tap water for additional 6 months.

Values are mean ± SD. Calculations based on: a). Kcal/g or mL: 3 (food), 0.42 (Cola) and 0.01 (Light cola); b). Na^+^ mg/g or mL: 2 (food), 0.075 (Cola or Light cola).

* p<0.05

** p<0.01

*** p<0.001 vs. W at the end of the corresponding period (i.e.: treatment or wash-out)

# p<0.01 vs. respective group after treatment.

### Biochemistry and insulin sensitivity

**Fig 1:**
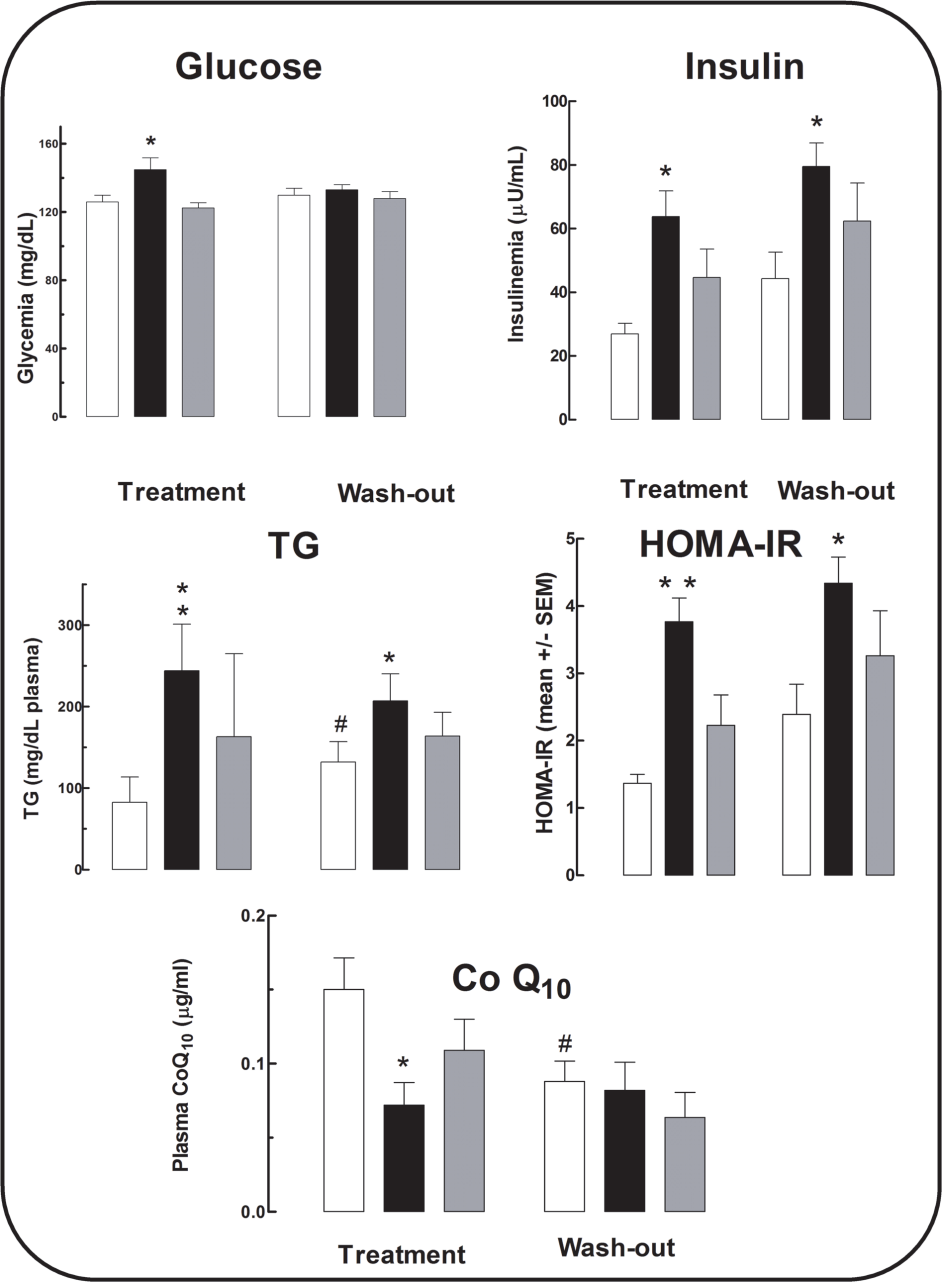
Blood chemistry, insulinemia, insulin resistance and Co Q10 levels. White columns: (W group), black columns: (C group), gray columns: (L group). Treatment period: months 0–6 of study. Wash out period: months 7–12 of study. *p<0.05, ** p<0.01 vs. W within period. For HOMA-IR *p< 0.004, **p<0.008 compared with W within the period. #p<0.05 in W between periods.

Regular cola drinking for 6 months (C_6_) resulted in hyperglycemia (+16%, F_2,18_ = 3.61, p<0.05) and irreversible hypertriglyceridemia (2.8-fold, F_2,18_ = 5.99, p<0.01) (i.e.: persistent after wash-out). An unexpected trend to hypertriglyceridemia was observed in L_6_ and L_12_ (Fig. 1).

Regular cola drinking resulted in irreversible hyperinsulinemia (2.4 fold in C_6_, p<0.01; 1.8 fold in C_12_, p<0.005) and HOMA-IR increase (2.7 fold in C_6_, p<0.008; 1.8 fold in C_12_, p<0.01) compared with age-matched W respectively.

### Oxidative metabolism

Plasma level of CoQ_10_ was lower in C_6_ compared with W_6_ (−52%,p<0.01). An unexpected trend to a decrease in CoQ_10_ level was observed in L group all over the study. Interestingly, age was associated with a decrease in plasma CoQ_10_ levels (−46% in W_12_ vs. W_6_) (Fig. 1).

### Quantitative morphology and immunohistochemistry

**Fig 2:**
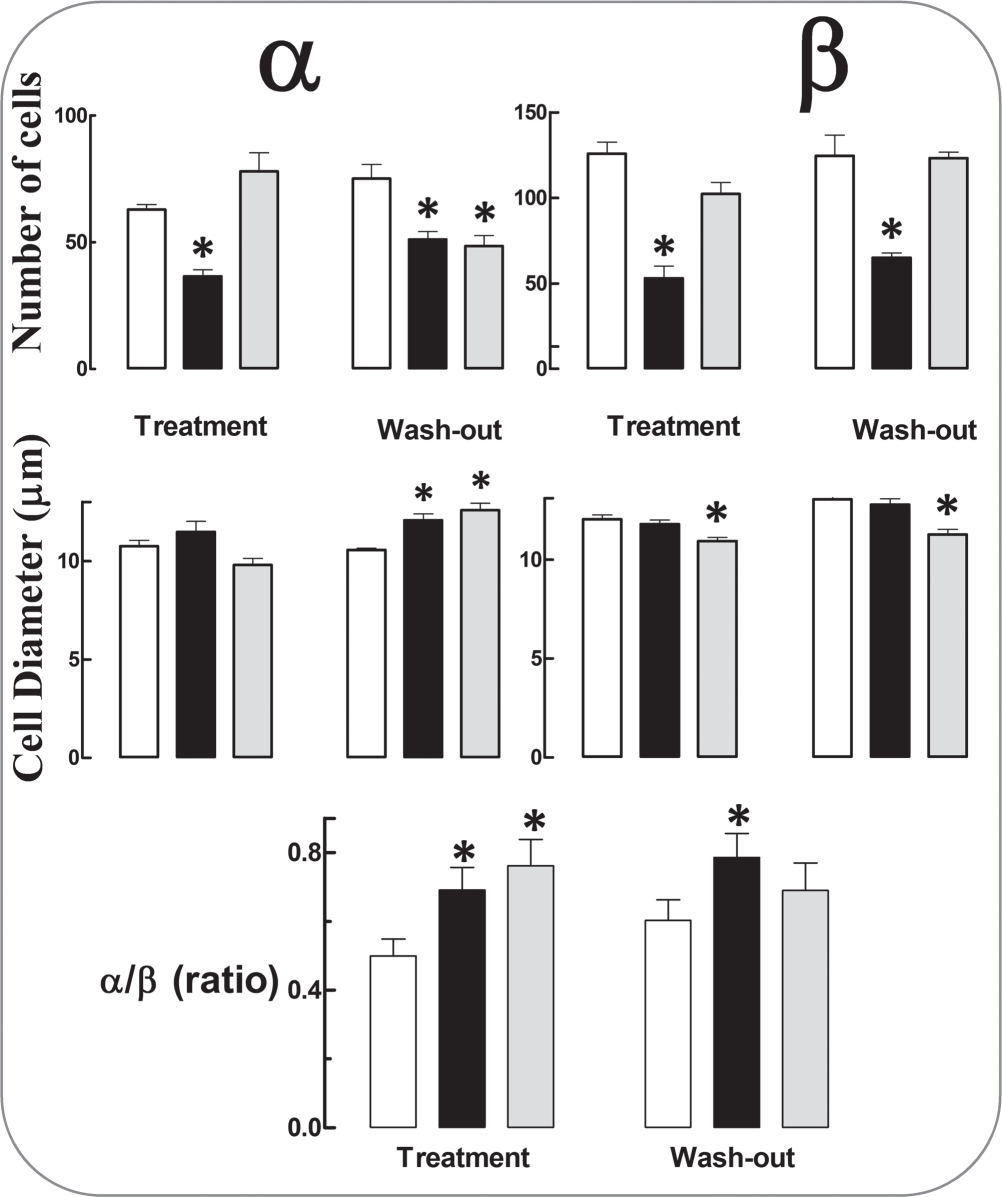
Alpha and beta cells in pancreatic islets: number, size and α/β-cell ratio. White columns (water, W group), black columns (regular cola, C group), gray columns (light cola, L group). Treatment period (months 0–6 of study). Wash out period (months 7–12 of study). Mean values ± SE are shown. *p<0.05 compared with W within the period.

Regular cola drinking caused an irreversible decrease in the number of both α cells (−42% C_6_, −32% C_12_,p<0.01) and β cells (−58% C_6_, −42% C_12_,p<0.001) and a moderate increase in the size of α cells after wash-out (+14%, p<0.001 C_12_, +7% NS, C_6_) (Fig. 2).

Light cola drinking affected β cells leading to irreversible reduction in size (−9% in L_6_, p<0.001; −14% in L_12_, p<0.0001) and affected α cells as well causing a 19% increase in size (p<0.0001) and a 35% reduction in number (p<0.01) only after wash-out (L_12_).

Accordingly, the α/β-cell ratio increased following consumption of either type of cola beverage. However, the effect of regular cola was irreversible (α/β-cell ratio = +38% in C_6_, +30% in C_12_, p<0.001vs. W_6_ and W_12_ respectively) while the effect of light cola was not (α/β-cell ratio = +52% in L_6_, p<0.001 vs. W_6_; +15% in L_12_, NS vs. W_12_).

Treatment with cola drinks did not substantially affect the size of the islet over the study time.

Insulin immunolabeling decreased: −59.7% in C_6_(p<0.00019, −50.3% in C_12_ (p<0.0001) and −33% in L_6_ (p<0.001) compared with age-matched W groups. Glucagon immunopositivity was lower in C_6_compared with W_6_(−33%, p<0.001).

Neither regular cola nor light cola drinking modified the nuclear expression of PCNA and caspase-3: isolated positive nuclei represented< 1% of the total population of insular cells. However, lower cytoplasmic immunopositivity for PCNA was found in C (−60% in C_6,_ −78% in C_12_, p<0.0001) and in L_12_ (−80%, p<0.0001) compared with age-matched W group.

Regular cola drinking strikingly increased the cytoplasmic expression of Trx1 (2.25-fold in C_6_ vs. W_6_; 2.72-fold in C_12_ vs. W_12,_ p<0.0001) and Prx2 (3-fold in C_6_ vs. W_6_; 2-fold in C_12_ vs. W_12,_ p<0.0001). Light cola drinking induced a remarkable increase in Trx1 (3-fold) and Prx2 (2-fold) after wash-out (p<0.0001, L_12_ vs. W_12_). Correlation was found between HOMA-IR and cytoplasmic expression of TRX1, Prx2 and PCNA(r, % mutually explained variation):0.839, 71%, p<0.037 for Trx1; 0.878, 77%, p<0.022 for Prx2; −0.893, 79%, p<0.017 for PCNA all over the study time (Table 2).

**Table 2:** Quantitative morphology and immunohistochemistry data.

	Treatment			Wash-out		
Group	W_6_	C_6_	L_6_	W_12_	C_12_	L_12_
**α/β-cell ratio**	0.4±0.1	0.8±0.3**	0.6±0.2*	0.4±0.1	0.7±0.2**	0.5±0.6
**A_islet_ (10^4^ μm^2^)**	2.2±0.4	1.8±0.6	2.42±0.9	2.5±0.7	2.3±0.4	2.5±0.7
**A_α_-cells (10^4^ μm^2^/islet)**	0.6±0.1	0.4±0.1*	0.6±0.2	0.7±0.2	0.6±0.1	0.6±0.2
**A_β_-cells (10^4^ μm^2^/islet)**	1.5±0.3	0.6±0.3**	1.0±0.3*	1.7±0.6	0.8±0.1**	1.3±0.1
**A_PCNA_ (10^4^ μm^2^/islet) (cytoplasmic)**	0.05±0.04	0.02±0.03**	0.05±0.06	0.04±0.02	0.009±0.006**	0.008±0.006**
**A_Trx1_ (10^4^ μm^2^/islet)**	0.4±0.3	0.9±0.1**	0.4±0.3	0.33±0.06	0.9±0.1**	1.0±0.1**
**A_Prx2_ (10^4^ μm^2^/islet)**	0.3±0.1	0.9±0.0**	0.2±0.1	0.4±0.1	0.8±0.1**	0.8±0.1**

Values are means ± SD. Differences were analyzed using the Kruskal-Wallis test and the Dunn´s Multiple Comparison test. A = area.

*p<0.001

**p<0.0001 vs. W within the period.

**Fig 3:**
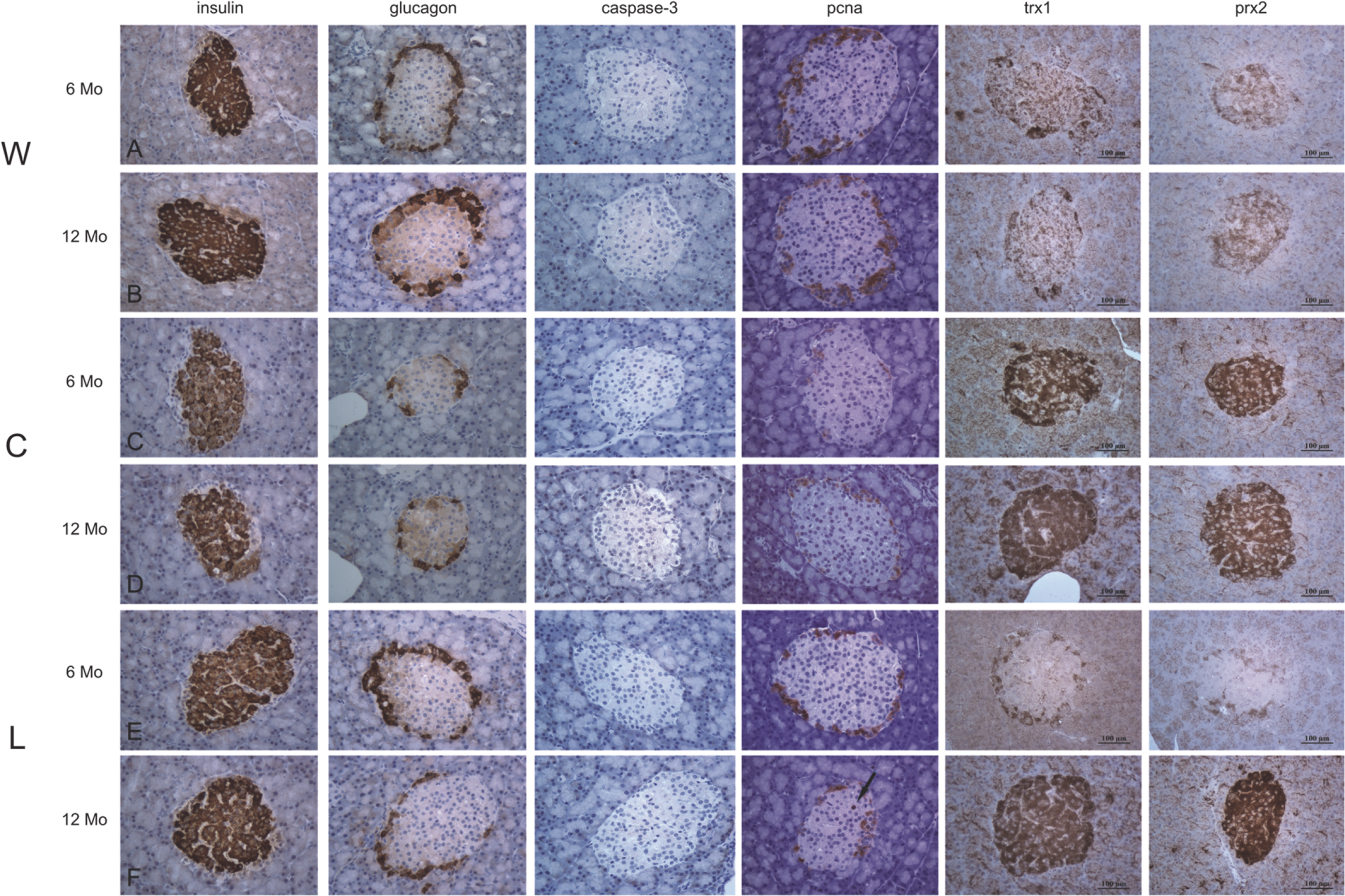
Representative immunolabeling for insulin, glucagon, caspase-3, PCNA, Trx1 and Prx2 in pancreatic islets in water (W), regular cola (C) and light cola (L) groups. **Longitudinal Panels A and B**: Classical cytoplasmic expression of insulin and glucagon was observed at 6 and 12 months of study. **Longitudinal Panels C and D**: The area of immunolabeling for insulin was irreversibly reduced after regular cola drinking. In addition, the cytoplasmic expression of glucagon showed a transient decrease at 6 months which recovered at 12 months. Regular cola drinking did not modify the apoptotic and proliferative activities. However, over the study time, the cytoplasmic immunolabeling for PCNA decreased and immunolabeling for Trx1 and Prx2 increased, suggesting a complex phenomenon linked to the redox pathway. **Longitudinal Panels E and F**: Light cola treatment induced a reversible decrease in insulin immunolabeling at 6 months and did not modify the cytoplasmic expression of glucagon. Scarce effects in apoptotic or proliferative conditions were observed after light cola drinking (the arrow indicates an isolated PCNA positive nucleus within the islet). Interestingly, cytoplasmic expression of PCNA decreased while cytoplasmic immunolabeling for Trx1 and Prx2 increased at the end of the wash-out period. Magnification 40 X. Scale bar: 100 μm. PCNA: Proliferating cell nuclear antigen; Trx1: thioredoxin 1; Prx2: peroxiredoxin 2.

Qualitative immunohistochemical findings are shown in Fig. 3.

## Discussion

Present findings support the impact of long-term chronic cola drinking on pancreas morphology and function. Pancreatic storage of insulin and glucagon decreased and β and α cells declined in number and size according to cola composition, i.e.: sugar-sweetened or artificially sweetened. Interestingly cola drinking raised the α/β-cell ratio regardless cola composition while neither change in proliferative nor apoptotic markers was observed.

The striking increase in triglycerides following regular cola consumption might be related to the high content of fructose in the drink.

Nuclear immunolabeling for caspase-3 and PCNA was very low, indicating negligible apoptotic and proliferative activity. The cytoplasmic expression of PCNA, specially observed at the islet periphery, might suggest other functions than synthesis and reparation of deoxyribonucleic acid (DNA) which is related to the nuclear expression of PCNA. Recent evidence indicates that cytoplasmic PCNA stimulates glycolysis via activation of glyceraldehyde-3-phosphate dehydrogenase [16]. Glutathione production could be indirectly reduced leading to oxidative stress as described in neurons [17]. An inverse pattern of immunolabeling for cytoplasmic PCNA, Trx1 and Prx2 was observed suggesting increased glutathione availability to thioredoxin and peroxiredoxin in this context. The hypothesis of an oxidative microenvironment is reinforced by correlation found for Trx1, Prx2 and cytoplasmic expression of PCNA with HOMA-IR. Glutathione and thioredoxins’ systems are known to act in concert and insulin resistance and diabetes are associated with decreased antioxidant capacity [18].

The decrease in α and β cells reported in this paper was not associated with less proliferation or enhanced apoptosis. Instead cells might have been spared by following differentiation and/or transdifferentiation routes [19]. The increase in α/β ratio might be explained by β-to-α transdifferentiation [20]. Alternatively α cell may be more resistant than β cell to the effects of cola drinking.

The concepts of β-cell dedifferentiation and transdifferentiation in diabetes are sometimes mistakenly confused [19]. Transdifferentiation is the direct conversion of one type of adult cell into an alternate type of cell with a different function [21] while dedifferentiation is the involution to an immature cell type. Accurate functioning of the pancreatic β cell is paramount to glucose homeostasis [22]. Understanding the mechanisms involved in β cell coping with stressful conditions is mandatory in order to clarify how β-cell dysfunction and islet remodeling contribute to diabetes.

Present findings are compatible with a glucotoxic loss of β cell function with depletion of insulin content. Multiple signaling pathways contribute to the adverse effects of glucotoxicity [23]. The increased expression of thioredoxins and the reduction in plasma levels of CoQ_10_ observed after cola drinking is consistent with an oxidative stress condition resulting from hyperglycemia. Hyperglycemia induces synthesis of reactive oxygen species by glucose oxidation, leading to an increased production of advanced glycosylation end products, as well as inflammation and oxidative stress[24]. On the other hand, artificial sweeteners readily originate advanced glycosylation end products with pro-oxidative and inflammatory effects. Failure to maintain a functional β-cell population is a serious problem [25]. When survival of overworking β cells is compromised, sparing the survivor cells may be a solution. Then, β cells may lose their mature identity and dedifferentiate to an insulin-negative neurogenin 3-positive stage (transcription factor neurogenin 3 is predominant during endocrine pancreas embryogenesis). Eventually, provided survival-threatening conditions disappear, dedifferentiated cells can evolve to mature insulin-positive β cells. Recent evidence demonstrates that β cell dedifferentiation, rather than apoptosis, is the main mechanism of loss of insulin-positive cells [26]. This mechanism may help explain the gradual decrease in β cell mass in long-standing diabetes and recovery of β cell function in type 2 diabetes following insulin therapy [26].

Remarkable plasticity has been reported in murine β cells which can largely adapt to altered insulin demand all over life-time [27]. The increase in α/β-cell ratio is interpreted as the result of reprogramming or transdifferentiation of β to α cell. Endocrine pancreas might cope with a compromising environment (oxidative stress, hyperglycemia) by triggering β and α mutual transdifferentiation, not only shortening the time delay to satisfy metabolic demand but reducing energy costs as well, an advantageous solution from the biological viewpoint [28].This is in agreement with recent findings pointing to the so called islet remodeling under critical situations in which insular endocrine cells should give fast response to a myriad of different metabolic situations. The possible existence of a β-α, α-β switch (or other cell phenotypes) is indeed more economic and faster than cell proliferation and it may sustain a chronically elevated hormone production.

The sweetener aspartame (L-aspartyl-L-phenylalanine methyl ester) and the caramel colorant are rich in advanced glycation end products that potentially increase insulin resistance and inflammation [2, 29]. During regular soft drinks consumption, fat accumulates in the liver by the primary effect of fructose which increases lipogenesis, and in the case of diet soft drinks, by the additional contribution of aspartame sweetener and caramel colorant. Aspartame before glucose ingestion augments glucagon-like peptide-1 (GLP-1) secretion and can contribute to obesity, insulin resistance and type 2 diabetes [30]. Aspartame is absorbed from the intestine and broken down to phenylalanine (50%), aspartic acid (40%) and methanol (10%) in the liver. Methanol is converted to formate, formaldehyde, diketopiperazine (a carcinogen) and a number of other highly toxic derivatives [31]. These and other issues have raised concern about the use of aspartame ever since its approval by the U.S. Food and Drug Administration in 1974.

This study shows that artificially sweetened cola may lead to health problems as well. The effects of light-cola drinking (striking increase in Trx1 and Prx2, a robust trend to hypertriglyceridemia, decrease in plasma level of CoQ_10_ and increase in HOMA-IR, reduction in β cells’ size, increase in α/β-cell ratio, increase in α cells’ size, decrease in α cells’ number) might suggest the development of an oxidative stress condition as well. Our findings are consistent with a recent publication showing that artificial sweeteners alter gut microbiota, induce glucose intolerance and increase susceptibility to metabolic disease [32]. Propionate, a bacterial end product of aspartame in the gut, is a highly gluconeogenic substrate and may contribute to increased susceptibility to metabolic disease [33].

In conclusion, we report evidence supporting that chronic cola consumption impairs pancreatic storage of insulin and glucagon, increases α/β-cell ratio and causes a striking increase in triglycerides and oxidative stress. This experimental model study opens new avenues to improve our knowledge of the metabolic syndrome and the associated decline in pancreatic function following long-term ingestion of cola drinks.
